# Evidence of Segregated Spawning in a Single Marine Fish Stock: Sympatric Divergence of Ecotypes in Icelandic Cod?

**DOI:** 10.1371/journal.pone.0017528

**Published:** 2011-03-07

**Authors:** Timothy B. Grabowski, Vilhjálmur Thorsteinsson, Bruce J. McAdam, Guđrún Marteinsdóttir

**Affiliations:** 1 Institute of Biology, University of Iceland, Reykjavík, Iceland; 2 U.S. Geological Survey, Texas Cooperative Fish and Wildlife Research Unit, Texas Tech University, Lubbock, Texas, United States of America; 3 Marine Research Institute (Hafrannsóknastofnunin), Reykjavík, Iceland; Roehampton University, United Kingdom

## Abstract

There is increasing recognition of intraspecific diversity and population structure within marine fish species, yet there is little direct evidence of the isolating mechanisms that maintain it or documentation of its ecological extent. We analyzed depth and temperature histories collected by electronic data storage tags retrieved from 104 Atlantic cod at liberty ≥1 year to evaluate a possible isolating mechanisms maintaining population structure within the Icelandic cod stock. This stock consists of two distinct behavioral types, resident coastal cod and migratory frontal cod, each occurring within two geographically distinct populations. Despite being captured together on the same spawning grounds, we show the behavioral types seem reproductively isolated by fine-scale differences in spawning habitat selection, primarily depth. Additionally, the different groups occupied distinct seasonal thermal and bathymetric niches that generally demonstrated low levels of overlap throughout the year. Our results indicate that isolating mechanisms, such as differential habitat selection during spawning, might contribute to maintaining diversity and fine-scale population structure in broadcast-spawning marine fishes.

## Introduction

It seems intuitive that populations of marine fishes should exhibit adaptations to their local environment that optimize performance. On the other hand, it is difficult to envision how such fine-scale population structure can be maintained in species with dispersive, planktonic life history stages inhabiting an open environment with few barriers to gene flow, such as that exemplified by most marine ecosystems [1]–[3]. Increasingly there are indications of local adaptations in marine fishes at spatial scales that cannot be explained solely by the complexities of oceanographic features acting as a dispersal barrier [1], [2], [4]. However, the ecological extent of these adaptations remains largely unexplored in the field. Given the high rates of exploitation experienced by many marine fishes and the challenges of large-scale climatic changes, understanding the extent of diversity contained within a management unit and their influence on population dynamics, connectivity, and resiliency is an essential prerequisite for effective management and conservation [5], [6].

**Figure 1:**
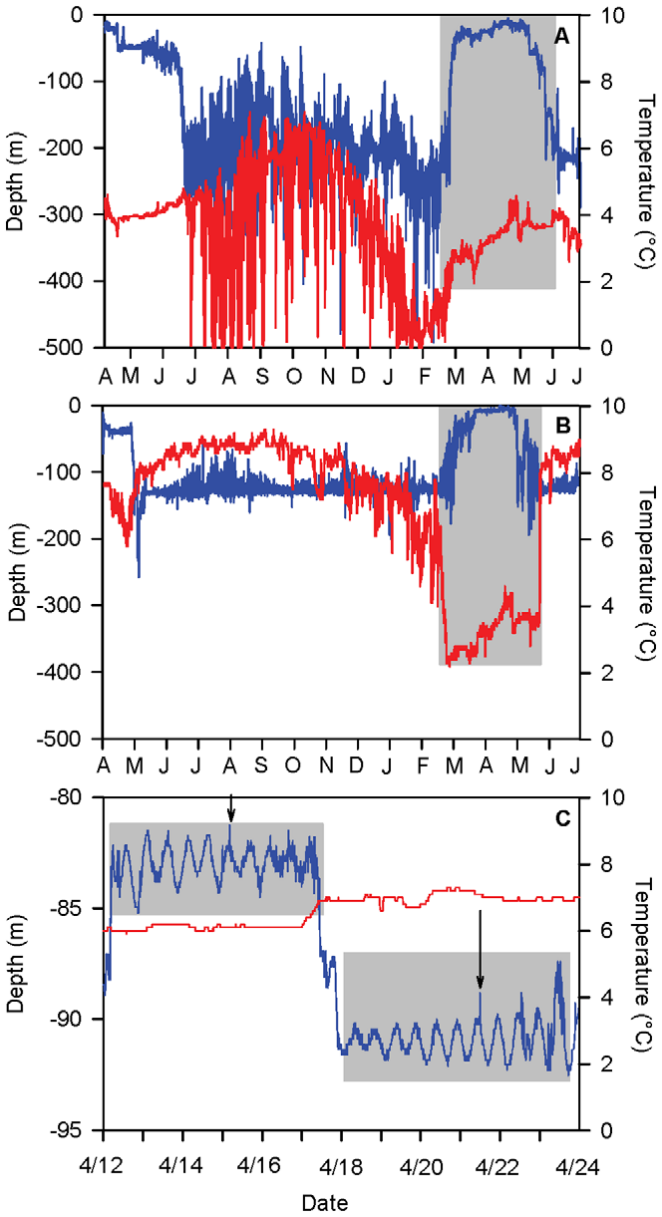
Temperature and depth profiles collected by electronic data storage tags implanted in Atlantic cod. Examples of one year depth and temperature profiles of frontal (A) and coastal (B) behavioral types of Atlantic cod in Iceland showing in-migration to spawning habitat, time spent at spawning grounds, and out-migration back to feeding grounds in the highlighted areas. Temperature is represented by a red line while depth is represented by a blue one. Periods interpreted as time spent in spawning aggregations was characterized by the highlighted portions with a clear tidal signature between in-migration and out-migration (C). Arrows indicate events interpreted as possible Atlantic cod spawning events as described by Brawn (1961a, b), Rose (1993), and Hutchings et al. (1999).

**Figure 2:**
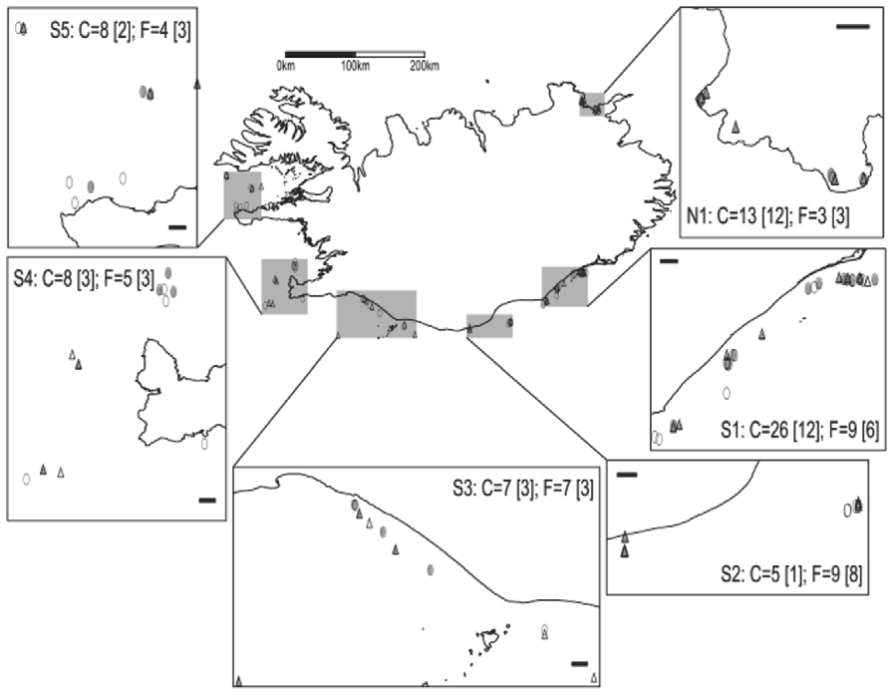
Original capture locations of Atlantic cod implanted with data storage tags (DSTs) around Iceland. Insets show the sampling regions in north (N1) and south (S1-S5) Iceland and indicate the number of recovered coastal (C, ○) and frontal (F, **▵**) individuals originally tagged and released in that area. The bracketed numbers and filled symbols represent individuals for whom spawning information could be extracted from their DST profile. Scale bar within each inset indicates a distance of 10 km.

In Atlantic cod *Gadus morhua*, perhaps the most intensively researched example of a broadcast spawning marine fish, local adaptations in life history traits have been noted amongst populations separated by relatively large distances [7]. More recently, population structure has been discovered at much finer scales than previously thought possible [1], [8]–[13]. Throughout its range in the North Atlantic, cod from different regions exhibit a high degree of diversification in life history traits such as growth rate and age at maturity [7], as well as behavior represented by repeated occurrences of migratory and non-migratory behavioral types within some of the Atlantic cod stocks [14]. In Iceland for example, genetic differentiation in the cod stock seems to be based on whether an individual spawns off northeastern or southwestern Iceland [15]. However, both of these populations have been shown to contain migratory and non-migratory individuals [16], [17] that may represent distinct ecotypes or even populations within the cod stock. The migratory cod have been described as a “frontal behavioral type” as they cross frontal boundaries, where warm Atlantic water meets cold, southward-flowing Arctic waters at the edge of the continental shelf to the southeast and northwest of the country. These cod exhibit a distinctive behavioral pattern of rapid vertical migrations between depths of 200–600 m while crossing steep thermal gradients during the feeding season [16; Figure 1]. In contrast, the non-migratory cod have been identified as a “coastal behavioral type” as they remain in relatively shallow water throughout the course of the year and rarely enter water deeper than 200 m [16; Figure 1] though still may make relatively long distance movements on the continental shelf. Coastal and frontal cod differ in their allele frequencies at the pantophysan (*Pan-*I) locus [17] and may show differences in their energy allocation strategies [18]. Despite these differences, both behavioral types return to the same general areas during spawning [16; Figure 2] and differentiation at neutral genetic loci has yet to be identified.

This is not an uncommon pattern as tagging recaptures have shown that even though the migratory and non-migratory cod exhibit differences in allele frequencies at the *Pan*-I locus [17], [19] and frequently other differences as well [7], [20], they are seemingly sympatric on the spawning grounds [7], [21], [22]. While it is unclear to what extent they interact with each other, it has been suggested that the two types segregate by depth during spawning [21] but other differences may exist in courtship or spawning behaviors as well. Furthermore, the extent of ecological variability between the behavioral types and its potential effect on their life history strategies has not been fully investigated. Electronic data storage tags (DSTs) offer great potential to resolve questions surrounding habitat use and behavior. In this study we used depth-temperature observations collected from DSTs to delineate ecological niches and establish that differences in seasonal niches influence habitat use during spawning periods.

## Results

As of spring 2008, 1488 Atlantic cod have been tagged with DSTs in Icelandic waters and to date approximately 31% have been recovered. Of the recovered cod, 104 individuals, representing approximately 6.37 million paired temperature and depth observations, were at liberty for a sufficient period of time to assess behavioral type and construct seasonal thermal-bathymetric niches. However only 57 individuals were at liberty for a sufficient period of time to determine the date and degree-days elapsed between out-migration from the spawning grounds and the initiation of in-migration and had profiles of sufficient quality to determine when spawning activity was initiated. The majority of the recovered individuals were from the southern population (*n* = 88), and the sexes were equally represented in each geographic population.

**Figure 3:**
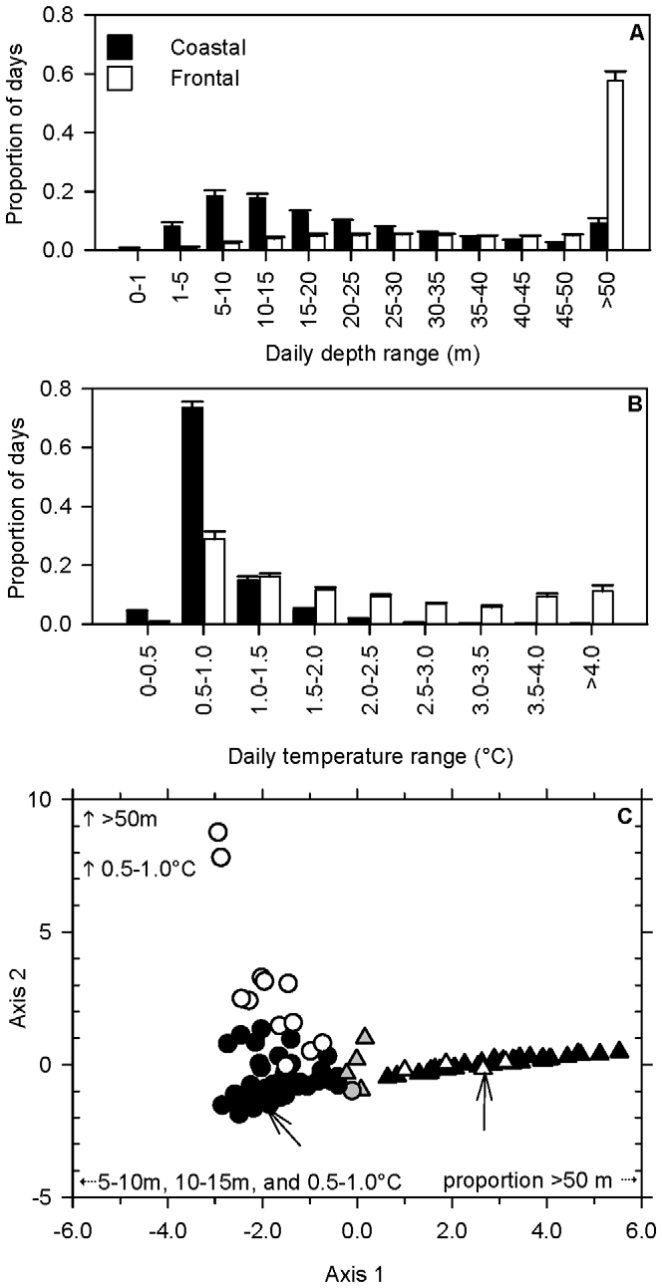
Definition of coastal and frontal behavioral types of Atlantic cod. Mean (± SE) proportion of daily depth (A) and temperature (B) ranges experienced by individuals tagged with electronic data storage tags (DSTs). Discriminant function analysis (C) performed on the proportion of daily depth and temperature ranges experienced by each individual indicated a clear separation between the coastal (northern: ○; southern: •) and frontal (northern: **▵**; southern: ▴) behavioral types based upon the proportion of depth and temperature ranges. The visual and DFA classifications of the five individuals represented by a gray circle or triangle could not be reconciled and these individuals were excluded from further analysis. Arrows indicate individuals whose depth and temperature profiles are displayed in Fig. 2a and 2b.

The coastal behavioral type comprised approximately two-thirds (*n* = 67) of the recovered individuals and no individuals were observed to alternate between coastal and frontal behavioral patterns during their time at liberty. The DFA identified two clusters corresponding to the coastal and frontal behavioral types and separated along the first canonical variable (R_c_^2^ = 0.911; *F*_15,276_ = 30.24; *P<*0.0001; Figure 3). The axis consisted primarily of an increasing proportion of days with a depth range >50 m and temperature range 2.5–3.0°C as well as a decreasing proportion of 0.5–1.0°C days. A second canonical variable described the variation within the behavioral types in terms of an increasing proportion of days with 1.0–5.0 m depth range and a decrease in the proportion of 5.0–10.0 m days (*R_c_* ^2^ = 0.760; *F*_8,202_ = 14.53; *P<*0.0001; Figure 3). There were not a large number of intermediate forms between the behavioral types as the visual and quantitative classifications could not be reconciled in only five individuals. These five individuals were excluded from further analysis.

Frontal cod inhabited deeper water than their coastal counterparts during 01 June – 31 December (*t*_71_ = 12.30; P<0.0001). However the behavior of individuals, as described by the first canonical variable, was largely independent of the mean depth occupied during this time (*F*_1,105_ = 1.93; *P* = 0.17). The behavior of coastal fish in deep (150–250 m) water was more similar to that of other coastal individuals inhabiting shallower water as described by daily depth and temperature ranges than frontal cod and vice versa. Likewise, the variability within behavioral types as described by the second canonical variable was independent of mean depth (*F*_1,105_ = 0.12; *P* = 0.73).

**Figure 4:**
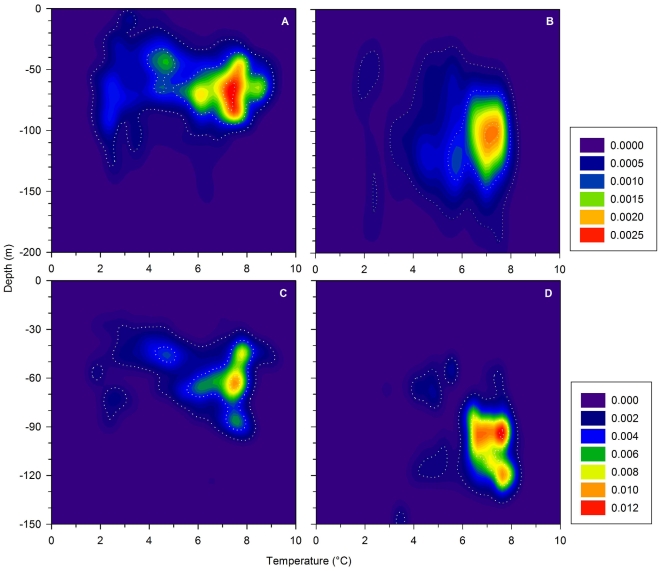
Bivariate kernel density estimates of thermal-bathymetric niches occupied during potential spawning activity of Atlantic cod. Thermal-bathymetric niches occupied during the spawning season as defined as the period between the end of in-migration and outmigration (A, B) and for periods of inferred presence in a spawning aggregation as indicated by the presence of a clear tidal signature during the spawning season (C, D) are presented for southern coastal (A, C) and frontal (B, D) behavioral types in the Icelandic stock of Atlantic cod. Dotted lines enclose the 95^th^, 50^th^, and 10^th^ percentiles of the distributions.

During the spawning season, southern frontal cod generally occupied deeper water than southern coastal cod (Figure 4). A modified version of Syrjala's test [23] was used to compare the thermal-bathymetric niches of southern coastal and frontal cod for two different definitions of the spawning season. The more inclusive and conservative definition encompassed the entire spawning season and used data from the period between the end of in-migration and outmigration. We also made the same comparison confining our analysis to periods when a clear tidal signature was present, which was interpreted as participation in spawning aggregations based on published accounts of cod spawning behavior [see Materials and Methods]. The difference between the thermal-bathymetric spawning niches between in and out migration of coastal and frontal fish was greater than that between randomized groups of fish (*n = *27 coastal, 26 frontal, 494,498 observations, *P*<0.001) despite being captured in close proximity while on the spawning grounds (Figure 2). Furthermore, southern coastal and frontal cod did not occupy the same thermal-bathymetric niches (Figure 4) when exhibiting a depth profile with a clear tidal signature (*n = *21 coastal, 23 frontal, 40,903 observations, *P*<0.001). The separation between coastal and frontal cod during these periods when a clear tidal signature was present seemed to be greater than that during the period between in and out migration (Figure 4). Northern coastal and frontal cod exhibited a very similar pattern despite being captured from within the same fjord system. However, a larger sample size is required to more effectively evaluate whether their distributions when engaged in a spawning aggregation are different (*n = *12 coastal, 3 frontal, 6,660 observations, *P* = 0.06).

**Figure 5:**
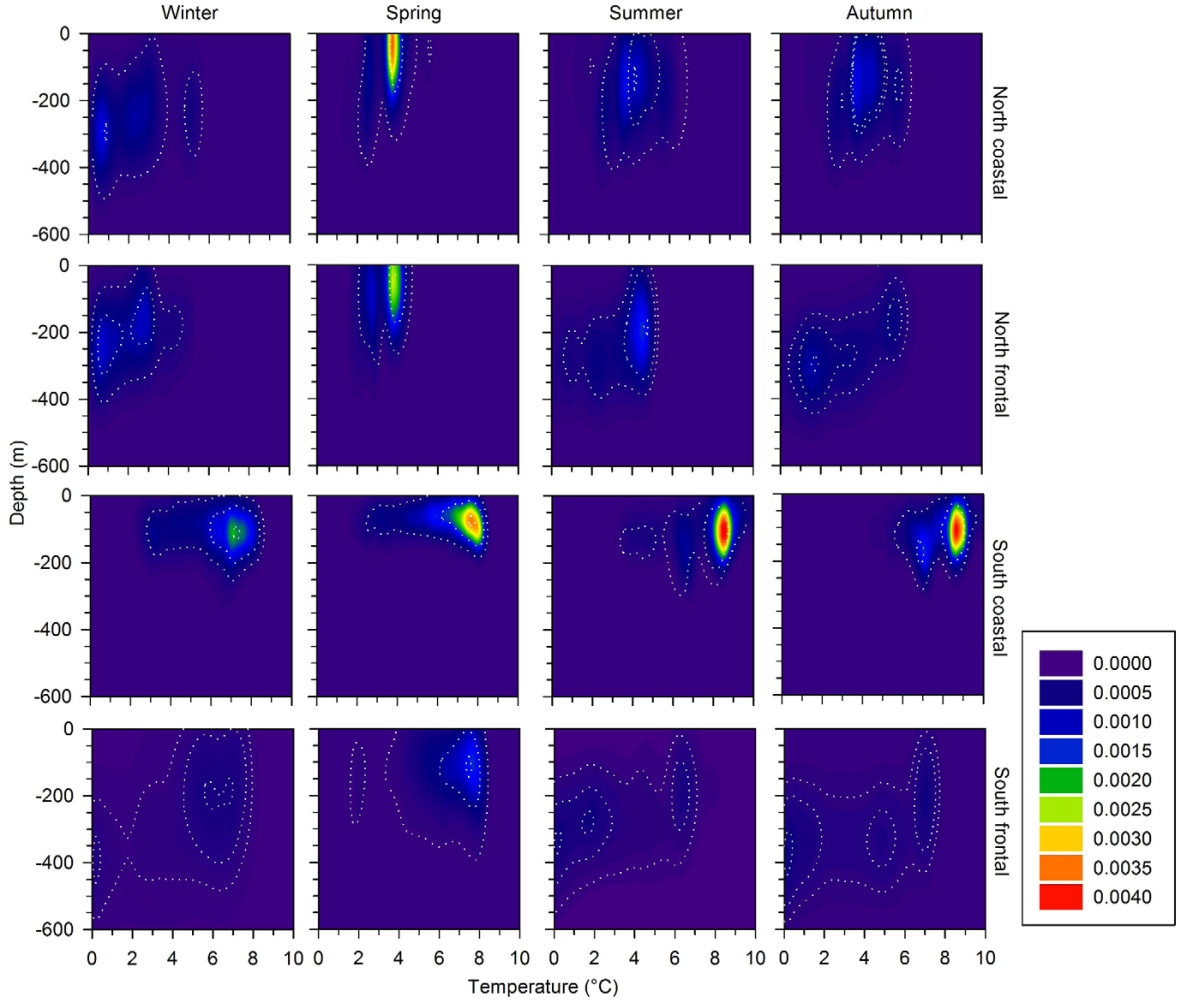
Bivariate kernel density estimates of seasonal thermal-bathymetric niches occupied by Atlantic cod ecotypes around Iceland. Dotted lines enclose the 95^th^, 50^th^, and 10^th^ percentiles of the distributions.

The separation in thermal-bathymetric niches was consistent throughout the remainder of the year for the southern population, as coastal cod generally inhabited a narrower seasonal range of temperatures and depths (Figure 5). However, there seemed to be a higher level of overlap between the behavioral types in northern cod (Figure 5). The same test was used to compare the seasonal thermal-bathymetric distributions of northern (*n = *12 coastal, 3 frontal; observations ≥70,848; *P*≤0.10) and southern fish (*n = *53 coastal, 35 frontal; observations ≥564,830; *P*≤0.001). After testing the eight null-hypotheses using the Holm-Bonferroni method, we found significant differences between the thermal-bathymetric niches of southern coastal and frontal cod in all seasons but only in winter and summer for northern coastal and frontal cod. There was no evidence that males and females of the same behavioral type occupied different thermal bathymetric niches (*P*≥0.43).

**Figure 6:**
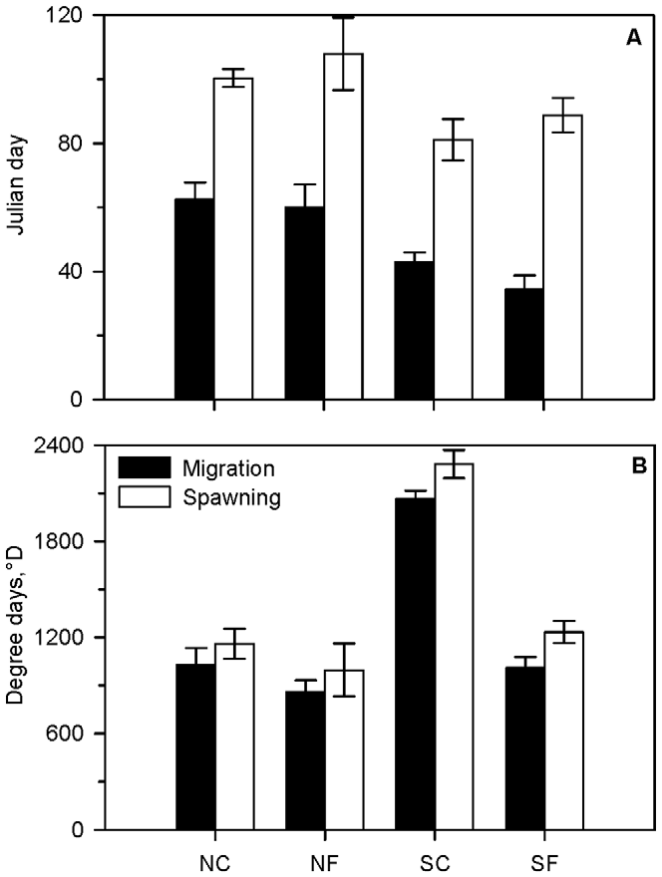
Phenology of migration and spawning of Atlantic cod in Iceland as extrapolated from recovered electronic data storage tags. Mean (± SE) Julian (A) and degree (B) day for initiating migration and spawning are shown for each Icelandic cod ecotype (NC = north coastal; NF = north frontal; SC = south coastal; SF = south frontal).

The seasonal thermal and bathymetric niches occupied by coastal and frontal cod seemed to be reflected in the phenology of migratory and spawning behavior (Figure 6). Regardless of behavioral type, southern cod initiated behavioral patterns consistent with participation in spawning aggregations at an earlier mean date than northern fish (*F*_1,61_ = 2.22; *P* = 0.02). Yet there were no differences in the mean start date for spawning or the initiation of migration between the behavioral types within a region (*F*_1,61_≤2.20; *P*≥0.14). There were no differences between northern coastal and frontal cod in the mean number of degree days accumulated prior to migration or spawning (*F*_1,61_≤0.50; *P*≥0.48), but again additional samples are required to confirm this conclusion. However, southern coastal cod accumulated a higher number of degree days prior to migration and spawning than frontal fish in the same region (*F*_1,61_ = 61.86; *P*≤0.0001). Southern frontal cod were more similar in to northern counterparts in terms of degree-day accumulation. Individuals at liberty for multiple years demonstrated a high level of interannual variability in the date and degree-day for the initiation of both migratory and spawning behavior. While there was no evidence of consistent differences related to sex, size, age, year, or site of capture, we lacked sufficient statistical power to definitively address these hypotheses due to small sample sizes of these groups within each population-behavioral type combination.

## Discussion

Atlantic cod in Icelandic waters seem to be diversified to behaviourally distinct ecotypes based on their occupation of different thermal and bathymetric niches. The behavioural patterns of coastal and frontal cod seem to be discrete and independent of the position of an individual on an ecological gradient such as depth. The associated differential habitat use separates coastal and frontal cod throughout the year and may be sufficient to isolate the behavioural types during spawning. Southern coastal and frontal cod occupy different thermal-bathymetric niches during the period encompassing the spawning season between in and outmigration. Greater separation between the behavioural types was observed during periods when clear tidal signatures were present. While we interpreted these periods as participation in a spawning aggregation, future research should focus on validating this interpretation. In either case, it seems that the behavioural types exhibit differential habitat selection during the spawning season in southern Iceland. Northern coastal and frontal cod exhibited a similar pattern despite using the same fjord system and being captured in close proximity (Figure 2) during the spawning season. The seasonal thermal-bathymetric niches of southern coastal and frontal cod are distinct throughout the year, but we found no such difference in spring and autumn for the northern fish. While this is an interesting finding, more data is needed from northern Icelandic cod to fully evaluate differences in thermal-bathymetric niches during spawning and throughout the remainder of the year among behavioural types.

The coastal and frontal behavioural types in northern and southern Iceland initiate migratory and spawning behaviour on similar dates. This is despite experiencing different thermal regimes that could potentially lead to different rates of gonadal development and maturation [24], [25] in the south and suggests that southern coastal and frontal cod may have diverged in more aspects than habitat selection and migratory and feeding behaviour. For example, the migratory and reproductive phenologies of the behavioural types may suggest physiological adaptations to their thermal niches. Despite the differences in thermal regimes and degree-days accumulated by the adult cod, there likely remains a selective pressure to time gonadal maturation and migration so that spawning occurs during environmental conditions favourable to larval survival [26], [27]. Alternatively, coastal and frontal Icelandic cod may show variation in their reproductive output or success that might be attributable to the differences in their thermal histories but cannot be assessed using DSTs. While the relationship between thermal history and reproductive physiology and phenology in Atlantic cod has been established [25], a better understanding of the ecological and population level implications of this relationship is needed.

The combination of temporal overlap and depth segregation observed between coastal and frontal Icelandic cod during spawning is comparable to that described for Norwegian coastal cod and northeast Arctic cod, which also exhibit a high degree of spatial and temporal overlap on the spawning grounds [21]. In both Iceland and Norway, the close proximity in which the different groups are encountered may be misleading. The habitat actually used for courtship and spawning by cod is likely a relatively small subset of what has been defined as cod spawning grounds by humans. For example, cod spawning grounds are defined for assessment purposes by the proportion of females in spawning condition present at a given location [28]. However, this may not be a particularly precise indicator for identifying locations where spawning is actively occurring [29] and the DST profiles seem to support this conclusion. While a period of 30–90 days may elapse between the end of in-migration and the initiation of out-migration, the depth profiles consistent with participation in spawning occur on average over only a 12-day period (range: 0–41 days). Cod seem to be present on what might be defined as a spawning ground for a longer period of time than that spent actively engaged in a spawning aggregation. A finer scale assessment of habitat use is needed to fully understand the extent of separation between coastal and frontal cod, particularly whether there is geospatial overlap of spawning aggregations. The relatively small amount of overlap seen in the thermal bathymetric niches during the spawning season or during participation in spawning aggregations (Figure 4) seems to be driven primarily by differences among the geographic regions (Figure 2). However, we did not have sufficient data to thoroughly evaluate this.

While the opportunity for interaction amongst adults might be limited by differential habitat use during spawning, it must be noted that coastal and frontal Icelandic cod could potentially mix during early life history [30] and thus may maintain a demographic connection between the behavioural types. It is unknown whether behavioural type is a genetically fixed trait or a phenotypic response to environmental factors [17], but the low numbers of individuals observed in this study exhibiting intermediate behavioural types and the lack of a strong relationship between depth and behaviour within coastal and frontal cod may be evidence for genetic determination. Furthermore, recent studies have noted the importance of post-settlement processes, such as natal homing and migratory behaviour, to the maintenance of population structure [31], [32]. These issues will need to be resolved in order to determine the precise nature and degree of interaction between the components of the Icelandic cod stock. However, our results are consistent with the presence of a complex population structure as previously concluded in studies of population genetic structure [15], physiology [16], [33], and otolith morphology and microchemistry [34], [35].

The Icelandic cod stock is not unique in its complexity, as various levels of differentiation have been reported within stocks throughout the range of Atlantic cod [1], [8]–[13], [19] in particular the repeated occurrence of migratory and non-migratory stock components [14]. While our results do not provide evidence for the origin of these strategies, they do suggest that these strategies are accompanied by behavioural and ecological differentiation, such as differential habitat selection, that might act as isolating mechanisms and allow a finer scale population structure than might otherwise be expected in a broadcast spawning marine fish. The question that then arises is whether this matters for the conservation and management of marine resources? It certainly emphasizes that the basic unit of management, the stock, is more a human construction based around geographic or political boundaries, than actual biological units [36]. A single management unit may feasibly contain numerous populations, each with their own ecological traits demonstrating varying degrees of demographic connectivity [2]–[4], [36]. In a conservation sense, this intraspecific diversity is important, providing long-term stability and resiliency for the species [5] as environmental conditions fluctuate. However it is also important for the management of commercial fisheries [5], [6], [37]; particularly if the stock components possess different life history characteristics such as growth rates, age at maturation, or fecundity. Managing for diversity does not necessarily run counter to effective management for economically viable fisheries [37]. Accounting for intraspecific diversity and local adaptations will only increase the chances of understanding and predicting population responses to fishing pressure, environmental impacts, and global climate change, and thus successful management efforts and sustainable fisheries [5], [6], [37]. It should no longer be assumed that all fish within a stock are equal and part of the same demographic and ecological unit. For example, there is evidence suggesting the decline of some stock components due to differences in vulnerability to exploitation can have serious repercussions to the stock as a whole [31], [38]. However if it can be properly accounted for and managed, the complexity found within the Icelandic cod stock and marine fish stocks generally could serve as the foundation for more sustainable fisheries capable of responding to natural and anthropogenic disturbances.

## Materials and Methods

### Fish tagging

We used DSTs (DST Centi and DST Milli: Star-Oddi Marine Device Manufacturing, Reykjavik, Iceland) capable of recording temperature in the range of −2.0–40.0°C (resolution: 0.032°C; accuracy: ±0.1°C) and depth in the range of 0–800 m (resolution: ±0.04% of measurement range; accuracy: ±0.4% of measurement range). The DST Centi possessed the battery life and data storage capabilities to sample temperature and depth at 10-minute intervals for up to three years, while the DST Milli tags were programmed to sample at 6-hour intervals June through February and 10-minute intervals March through May for two years.

Atlantic cod were sampled from spawning grounds around Iceland from 2002 through 2008 using gill nets and Danish seines (Figure 2). Individuals displaying no indication of barotrauma or external injury were selected for tagging. A DST was surgically implanted into each individual's abdominal cavity following the procedures outlined by Thorsteinsson and Marteinsdóttir [39]. The entire procedure was completed in less than five minutes and the fish was returned to the sea immediately upon its completion. Fish tagging activities conducted by V.T. under license number 0304-1901 issued by the Icelandic Committee for Welfare of Experimental Animals, Chief Veterinary Office at the Ministry of Agriculture, Reykjavik. Tagged fish were recovered by commercial fishermen.

### DST interpretation and data collection

Individuals were classified as coastal or frontal behavioural types visually from DST depth and temperature profiles based on the criteria described by Pálsson and Thorsteinsson [16] and Pampoulie et al. [17] and quantitatively using discriminant function analysis (DFA). Briefly, we examined the temperature and depth profile for 1 June-31 December to determine behavioural type for each individual. This time period was selected to eliminate any effects migratory or reproductive behaviour might have on the assignment of behavioural type. The classification of behavioural type is based on depth-temperature profiles outside the spawning season, and hence when testing for differences in spawning behaviour we are not making a circular argument based on the prior categorisation Individuals that showed a clear seasonal temperature signal over the course of the year and did not spend significant periods of time over the course of their time at liberty below 200 m (>10%) were visually classified as coastal cod, while those found below 200 m for the majority of the year and showed a highly variable temperature signal were visually classified as frontal cod (Figure 1). To assess these classifications quantitatively, we determined the daily temperature and depth ranges during 1 June-31 December. The daily ranges for each individual were sorted into twelve 5.0-m bins for depth (<1 m, 1–5 m, 5–10 m, …, 45–50 m, >50 m) and nine 0.5°C bins for temperature (0.0–0.5°C, 0.5–1.0°C, …, 3.5–4.0°C, >4.0°C) then the proportion of days accounted for by each bin was determined. These proportions were used in DFA to quantitatively assess the behavioural types assigned visually.

The date of out-migration from the spawning ground, in-migration back to the spawning grounds, and initiation and duration of spawning activity were visually identified for each individual from data collected by the DSTs using SeaStar v. 3.7.9.4 (Star Oddi Marine Device Manufacturing, Reykjavik, Iceland). Periods of migration were characterized by a period of consistent, directed change in the depth occupied by an individual usually initiated in late winter or early spring for in-migrations and late spring to midsummer for out-migrations (Figure 1a–b). The initiation of spawning behaviour was characterized as the point after in-migration when vertical movement was minimized and a clear tidal signature was evident (Figure 1c) consistent with cod behaviour during participation in a spawning aggregation as described in the literature [40]–[44]. Published descriptions of cod spawning behavior in both laboratory and field settings suggest that they form aggregations on or near the substrate [40]–[45] and that individuals, particularly males, remain in these aggregations for extended periods of time [45]. The presence of a clear tidal signature in the depth profile is indicative of an individual maintaining a more or less constant depth through the tidal cycle [46] as might be expected for an individual holding its position within a spawning aggregation. Most individuals exhibiting these periods with a clear tidal signature during a spawning season actually had depth profiles with several distinct periods interspersed with episodes of unclassified vertical movement. The DSTs also may have recorded rapid vertical movements during these periods with clear tidal signatures (Figure 1c) that may have indicated participation in a spawning event consistent with published descriptions [42], [44]. However due to the uncertainty of this interpretation and the temporal resolution of the data, only the periods where a clear tidal signature was evident were considered for analysis of niche use during spawning.

Elapsed time between out-migration and both in-migration and initiation of spawning was determined and used to calculate the degree-days (°D) between these life cycle events. The degree-day is a useful method of standardizing the effect of temperature on growth, development, and the timing of life-history events across individuals of poikilothermic organisms that may experience different temperature regimes [24]. A threshold temperature (T_o_) of 0.0°C was used for all degree-day calculations. Daily measures of °D were then summed for the periods of interest.

### Data analysis

Five different thermal-bathymetric niches were obtained for each fish. These were the sets of temperature-depth pairs occupied by the fish during the winter (December-February), spring (March-May), summer (June-August) and autumn (September-November) seasons, and during periods when individuals exhibited behavioural patterns consistent with participation in a spawning aggregation as described above. We tested whether the coastal and frontal fish occupied different niches using a variation of Syrjala's test [23] for difference between the spatial distributions of two populations. The test firstly normalises the number of observations at an individual and group level to remove any differences caused by sample size. A test statistic is computed by summing the squares of differences in abundance of observations between the two groups within rectangular regions of the niche space. In contrast to Syrjala's original method, our rectangles are randomly sampled from the space because data are not obtained from fixed spatial stations. The distribution of test statistics for data that meets the null hypothesis that there is no difference between the groups is obtained by repeatedly randomly allocating fish into two groups and computing a test statistic. The *P*-value for the null hypothesis is the quantile position of the actual test statistic within the set of values for randomised data. In every test, 2,000 rectangles were sampled to calculate the test statistic and 2,000 random permutations of the data were used to create the distribution. These numbers were comparable to those typically used in the unmodified Syrjala test and yielded consistent results with our data. Two distinct hypotheses were tested, firstly that the thermal-bathymetric niches occupied by coastal and frontal cod during spawning differed within both regions (north and south), and secondly that the niche space distributions differed between coastal and frontal fish in each region and season. In both cases the significance of *P*-values was tested using the Holm-Bonferroni method to account for multiple comparisons. A two-way multivariate analysis of variance (MANOVA) was used to evaluate differences in the Julian day and degree-day of the initiation of migration and spawning between region and behavioural type.
